# Mutant WDR45 Leads to Altered Ferritinophagy and Ferroptosis in β-Propeller Protein-Associated Neurodegeneration

**DOI:** 10.3390/ijms23179524

**Published:** 2022-08-23

**Authors:** Sokhna Haissatou Diaw, Christos Ganos, Simone Zittel, Kirstin Plötze-Martin, Leonora Kulikovskaja, Melissa Vos, Ana Westenberger, Aleksandar Rakovic, Katja Lohmann, Marija Dulovic-Mahlow

**Affiliations:** 1 Institute of Neurogenetics, University of Lübeck, 23562 Lübeck, Germany; 2 Department of Neurology, Charité—University Medicine, 10117 Berlin, Germany; 3 Department of Neurology, University Medical Center Hamburg-Eppendorf, 20251 Hamburg, Germany

**Keywords:** WDR45, BPAN, autophagy, ferritinophagy, NCOA4, ferroptosis, GPX4, GBA

## Abstract

Beta-propeller protein-associated neurodegeneration (BPAN) is a subtype of neurodegeneration with brain iron accumulation (NBIA) caused by loss-of-function variants in WDR45. The underlying mechanism of iron accumulation in WDR45 deficiency remains elusive. We established a primary skin fibroblast culture of a new BPAN patient with a missense variant p.(Asn61Lys) in *WDR45* (NM_007075.3: c.183C>A). The female patient has generalized dystonia, anarthria, parkinsonism, spasticity, stereotypies, and a distinctive cranial MRI with generalized brain atrophy, predominantly of the cerebellum. For the functional characterization of this variant and to provide a molecular link of WDR45 and iron accumulation, we looked for disease- and variant-related changes in the patient’s fibroblasts by qPCR, immunoblotting and immunofluorescence comparing to three controls and a previously reported WDR45 patient. We demonstrated molecular changes in mutant cells comprising an impaired mitochondrial network, decreased levels of lysosomal proteins and enzymes, and altered autophagy, confirming the pathogenicity of the variant. Compared to increased levels of the ferritinophagy marker Nuclear Coactivator 4 (NCOA4) in control cells upon iron treatment, patients’ cells revealed unchanged NCOA4 protein levels, indicating disturbed ferritinophagy. Additionally, we observed abnormal protein levels of markers of the iron-dependent cell death ferroptosis in patients’ cells. Altogether, our data suggests that WDR45 deficiency affects ferritinophagy and ferroptosis, consequentially disturbing iron recycling.

## 1. Introduction

Mutations in the *WDR45* (WD repeat domain 45) gene cause beta-propeller protein-associated neurodegeneration (BPAN, OMIM 300894), a subtype of a heterogeneous group of rare, monogenic disorders referred to as neurodegeneration with brain iron accumulation (NBIA) [1,2]. Based on numbers in the International Registry for NBIA and Related Disorders, 40–45% of all NBIAs are due to BPAN [1]. BPAN patients usually present with global developmental delay and autistic features in early childhood and subsequent regression in early adulthood with ataxic gait, hypotonia, mild spasticity, progressive dystonia-parkinsonism, anarthria and aphonia, as well as dementia [1]. Even though *WDR45* is localized on the X chromosome, BPAN does not clearly follow an X-linked dominant inheritance: both genders have similar clinical features; however, females are affected more frequently. Female patients usually harbor de novo, often truncating, germline mutations. These changes are predicted to lead to non-functional proteins and are presumed to be lethal for male embryos. Somatic mosaicism for pathogenic *WDR45* variants is the disease cause in affected boys [3]. Postmortem brain analyses in patients with *WDR45* mutations showed iron deposition primarily in the *substantia nigra* [4].

The WDR45 protein is involved in diverse cellular functions such as autophagy, cell cycle progression, transcriptional regulation, and signal transduction [5]. It binds to PI3P (phosphatidylinositol 3-phosphate)-enriched membranes at the ER (endoplasmic reticulum) and, together with PI3P, regulates autophagosome size and maturation [6,7]. Defective autophagic flux has been shown in *WDR45*-mutant patient-derived lymphoblastoid cells [2] and *wdr45*-knockout mice [6,8]. Furthermore, in accordance with the hypothesis of an underlying autophagic defect in BPAN, induction of autophagy has been shown to reduce the elevated iron levels and rescue the phenotype in *WDR45*-mutant patient-derived fibroblasts and neurons [9]. However, the molecular mechanisms explaining how decreased autophagy causes iron accumulation in WDR45 deficiency remain elusive.

Given that iron is both essential (e.g., as a co-factor of many enzymes) and potentially harmful (via formation of reactive oxygen species (ROS)) in cells, the proper homeostasis of this metal is vitally important for cells. Iron is mainly stored in cytosolic ferritin, a multimeric protein that compartmentalizes iron into a soluble non-reactive form, protecting cells from ROS [10]. In part, iron recycling is mediated through ferritinophagy, an autophagic turnover of ferritin. This process involves an autophagic cargo receptor, nuclear coactivator 4 (NCOA4), which interacts directly with ferritin and guides ferritin to the lysosome for degradation and iron release [11,12,13]. In addition to cytosolic ferritin, iron is stored in iron-rich organelles, including lysosomes and mitochondria [5]. Increased iron levels can directly damage mitochondria by oxidizing lipids and proteins via ROS and eventually cause mitochondrial degradation. Recently, it has been shown that loss of WDR45 increases cellular iron levels and oxidative stress and is accompanied by mitochondrial abnormalities and lysosomal dysfunction [9]. Moreover, ferritinophagy contributes to the initiation of ferroptosis (iron-dependent, non-apoptotic cell death) through the degradation of ferritin, which triggers labile iron overload, lipid peroxidation, membrane lesion, and finally, cell death [14,15,16]. Iron dysregulation has long been recognized as a cause of neuronal damage in iron-sensitive brain regions. Elevated iron levels have been associated with sporadic neurodegenerative diseases like sporadic Alzheimer’s disease (AD) or Parkinson’s disease (PD) [17,18]. Nevertheless, iron dyshomeostasis within susceptible brain regions is still insufficiently understood.

In this study, we demonstrate these previously reported molecular alterations in cells harboring another WDR45 mutation and expand the knowledge by unraveling the link between WDR45 mutations, ferritinophagy, and ferroptosis.

## 2. Results

### 2.1. Clinical Presentation and Genetic Analysis of Patient 1 Harboring a Recurrent Mutation in WDR45

**Figure 1:**
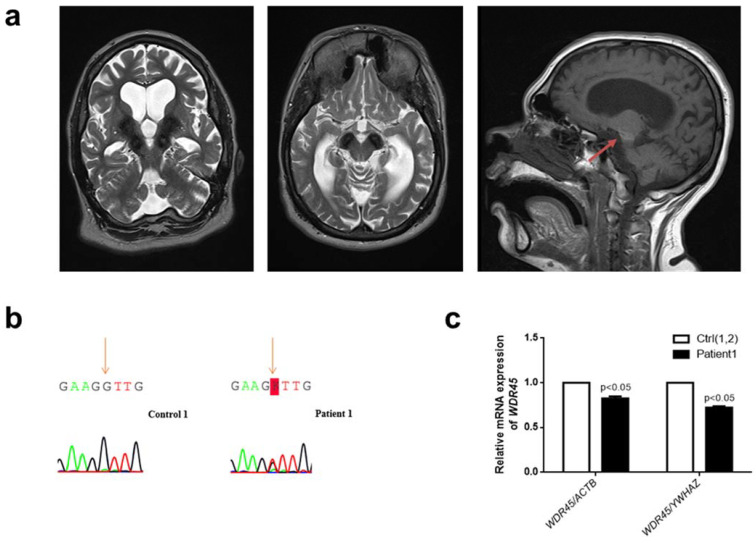
**Clinical and genetic analysis of a novel female patient (Patient 1) with a missense *WDR45* variant.** (**a**) Brain MRI from Patient 1. The *globus pallidus* and the *substantia nigra* are extensively hypointense bilaterally on axial T2 sequences indicating high levels of iron deposition. The characteristic BPAN T1-hyperintense halo surrounding the *substantia nigra* is also noted (red arrow). (**b**) Electropherograms of Sanger sequencing of complementary DNA (cDNA) from cultured fibroblasts from Patient 1 (right panel) and a healthy individual (Control 1) (left panel) in the reverse direction. The variant is highlighted: “K” represents “G” or “T.” (**c**) The quantification of *WDR45* mRNA levels in fibroblasts from Patient 1 compared to fibroblasts from two healthy individuals (Control 1 and Control 2—Ctrl(1,2)). Means and standard error of the mean (SEM) are indicated. Quantification is shown with the mean control level set as 1. The results are based on the mean ratios of *WDR45* expression compared to two reference genes, *ACTB* and *YWHAZ*. Statistical significance was analyzed by one-way ANOVA (*p* < 0.05 refers to a significant difference between Patient 1 and two healthy controls (Ctrl(1,2)).

We here report a 31-year-old female admitted to the University Hospital Eppendorf due to increasing slowness and poor balance. Since the age of 2 years, she has had focal seizures. Additionally, she has a history of delayed motor development, intellectual impairment, and poor speech production. Gait has always been difficult, with toe walking during early childhood. She has always been reluctant to engage in social interactions and only had restricted interests, often exhibiting repetitive behaviors, such as body rocking and purposeless to-and-from movements of the arms (Video S1). Over the past two years, symptoms have worsened significantly, with an overall reduction in motor output and repeated falls. Her treatment on presentation consisted of Lamotrigine (200-0-200) and Levetiracetam (750-0-1000). On neurological examination, the patient was anarthric with saccadic hypometria and clear hypomimia, spasticity with increased deep tendon reflexes, and bilateral extensor plantar responses. Generalized dystonia and bradykinesia were also noted. In addition, there was apparent leg weakness during gait with markedly reduced stride length and shuffling. Further, motor stereotypies with to-and-from movements of the arms were observed. Cranial MRI revealed generalized brain atrophy, predominantly of the cerebellum. There were extensive pallidal and nigral T2-hypointensities and a characteristic T1-hyperintense halo surrounding the *substantia nigra* (Figure 1a). The medical history and the clinical presentation of this patient with generalized dystonia with anarthria, Parkinsonism, spasticity, and stereotypies alongside the distinctive cranial MRI led to the suspected diagnosis of BPAN. Treatment was initiated with a daily dosage of 450 mg levodopa, which improved motor function and overall motivation. Within a follow-up period of two years, a stable effect of dopamine was noted, and no dyskinesias were observed.

Sequencing of blood genomic DNA revealed a previously reported [3] missense variant in WDR45 (NM_007075.3: c.183C>A; p.(Asn61Lys)) and thus confirmed the diagnosis of BPAN in our patient. Her parents were unavailable for testing. The variant was found neither in 1000 Genomes nor in GnomAD. According to ACMG criteria [19], the variant was classified as likely pathogenic. Sequencing of the *WDR45* cDNA confirmed the expression of the variant (Figure 1b). Quantitative PCR revealed significantly decreased *WDR45* gene expression levels in Patient 1 compared to two controls (*p* < 0.05) (Figure 1c). Consequently, this finding suggested lower levels of the WDR45 protein in our patient (~20–25%).

### 2.2. WDR45-Mutant Fibroblasts Exhibit Diminished Lysosomal Integrity and Altered Mitochondrial Network

**Figure 2:**
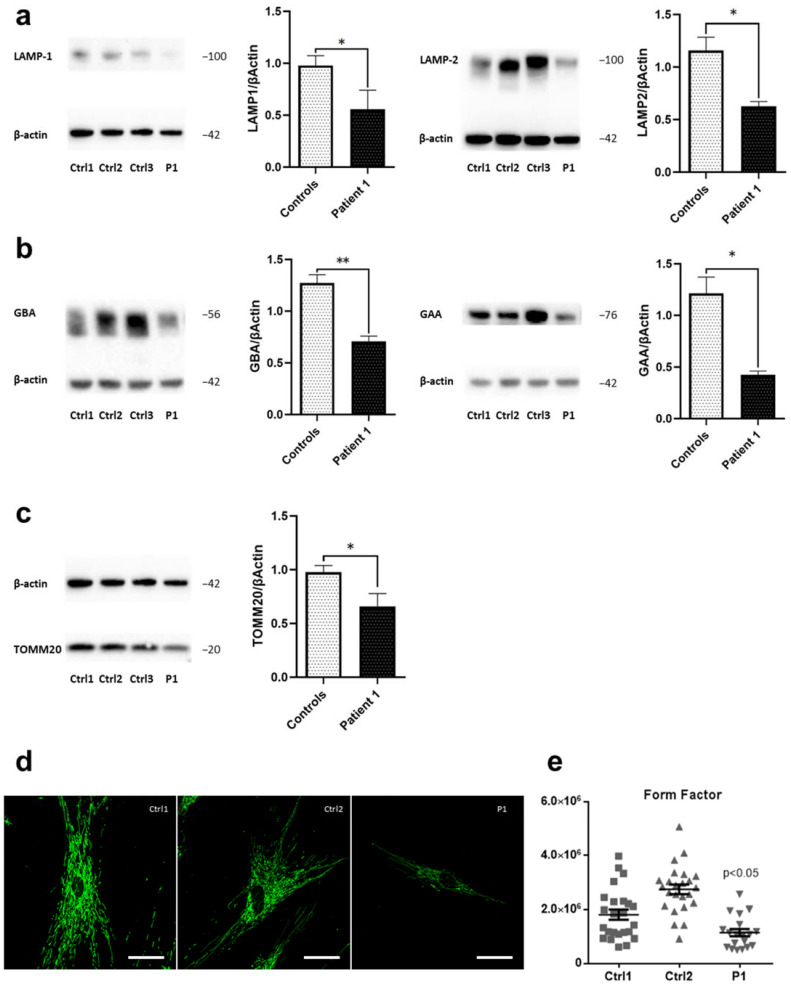
**Disrupted lysosomal and mitochondrial integrity in *WDR45*-mutant fibroblasts.** Western blot analysis of total protein extract from: (**a**) fibroblasts from WDR45 mutation carrier (P1—Patient 1) and three healthy controls (Ctrl1, Ctrl2, Ctrl3) with antibodies against the lysosomal proteins LAMP-1 (left panel) and LAMP-2 (right panel), and β-actin (loading control), (**b**) with antibodies against lysosomal enzyme GBA and β-actin (loading control) (left panel) and with antibodies against lysosomal enzyme GAA and β-actin (loading control (right panel). (**c**) Patient 1’s fibroblasts and three healthy controls with antibodies against the mitochondrial protein TOMM20 and β-actin (loading control). Data analysis was carried out for all Western blots with Ctrl1 set as 1. The error bars indicate standard error of the mean of n ≥ 3 independent experiments. Statistical significance was analyzed by Unpaired *t*-test (* *p* < 0.05; ** *p* < 0.01). (**d**) The mitochondrial network was visualized by confocal microscopy in fixed cells immuno-stained with anti-GRP75 (green). The scale bar corresponds to 100 μm. (**e**) A mean form factor was calculated as a measure of mitochondrial interconnectivity by using ImageJ (NIH software). Each dot represents the value from a single cell (15–20 cells per cell culture), and the mean and the error bar (SEM) per individual are indicated. Statistical significance was analyzed by one-way ANOVA (*p* < 0.05 refers to a significantly decreased form factor in Patient 1’s fibroblasts compared to healthy controls).

Based on the fact that increased amounts of iron are known to accumulate in lysosomes either as aggregated iron-containing proteins, damaged mitochondria, or due to the accumulation of iron-rich non-degradable materials (e.g., lipofuscin) [20], we first examined lysosomal function in patient-derived fibroblast cells and three healthy controls. We detected decreased levels of lysosomal markers LAMP-1 and LAMP-2 in Patient 1 compared to controls (Figure 2a).

Next, we analyzed protein levels of two lysosomal enzymes, glucosylceramidase beta/β-glucocerebrosidase (GBA) and acid α-glucosidase (GAA). GBA is an essential enzyme for lysosomal storage, catalyzing the hydrolysis of glucosylceramide. Variants in this gene have been associated with Parkinson’s disease [21]. Compared to healthy controls, the fibroblasts from Patient 1 showed significantly decreased GBA protein levels (Figure 2b, left panel). Moreover, fibroblasts from Patient 1 displayed lower levels of GAA, an enzyme responsible for the lysosomal hydrolysis and clearance of glycogen [22], in comparison to controls (Figure 2b, right panel). By this, we further confirmed the alteration of lysosomal function in WDR45 deficiency.

Apart from lysosomes, mitochondria represent one of the prominent cellular storage organelles for iron [5]. Of note, GBA depletion, which we registered in Patient 1’s fibroblasts, has been known to trigger mitochondrial dysfunction by inhibiting mitochondrial priming, a critical step for the selective removal of dysfunctional mitochondria by a process known as mitophagy [23]. Therefore, we next analyzed protein levels of the mitochondrial translocase TOMM20, located in the outer membrane, by Western blotting. TOMM20 protein levels were decreased in *WDR45*-mutant fibroblasts from Patient 1 compared to healthy controls (Figure 2c).

We further analyzed the effect of mutant WDR45 on the integrity of the mitochondrial network by calculating the form factor in *WDR45*-mutant fibroblasts and controls. The analysis demonstrated the expected, decreased mitochondrial branching and interconnectivity in *WDR45*-mutant fibroblasts from Patient 1 (Figure 2d,e) when compared to controls.

### 2.3. WDR45-Mutant Fibroblasts Exhibit Altered Autophagy

**Figure 3:**
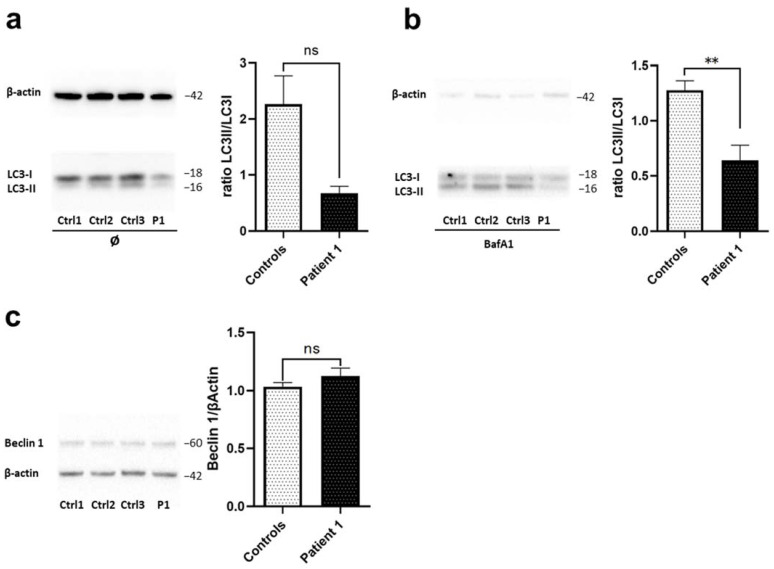
***WDR45*-mutant fibroblasts exhibit altered autophagy.** Western blot analysis of total protein extract from fibroblasts from: (**a**) *WDR45* mutation carrier (P1-Patient 1) and three healthy controls (Ctrl1, Ctrl2, Ctrl3) with antibody against the autophagosome marker LC3-II (membrane-bound form), and β-actin (loading control) under basal conditions, (**b**) and upon treatment with 10 nM of Bafilomycin A1 for 6 h. (**c**) Patient 1’s fibroblasts (P1) and three healthy controls (Ctrl1, Ctrl2, Ctrl3) with antibodies against Beclin 1 and β-actin (loading control). Data analysis was carried out for all Western Blots with Ctrl1 set as 1. The error bars indicate the standard error of the mean of n ≥ 3 independent experiments. Statistical significance was analyzed by Unpaired *t*-test (ns refers to *p* ≥ 0.05; ** *p* < 0.01).

Previous studies have shown defective autophagic flux in *WDR45*-mutant patient-derived lymphoblast cells [2] and *wdr45*-knockout mice [6,8]. Similarly, autophagosome synthesis occurred at a lower basal level in *WDR45*-mutant fibroblasts from another BPAN patient, Patient 2 [9]. Therefore, we next investigated the levels of autophagic marker proteins by immunoblotting. We observed seemingly (not significantly) decreased levels of the autophagosome marker LC3-II, a lipidated membrane-bound form of LC3, in *WDR45*-mutant fibroblasts from Patient 1 (Figure 3a) when compared to controls. Therefore, next we investigated autophagic flux by monitoring the effect of the lysosomal inhibitor (H+-ATPase inhibitor) Bafilomycin A1 (Baf A1) on LC3-II levels in *WDR45*-mutant fibroblasts from Patient 1. Baf A1 blocks LC3-II degradation, thus allowing one to estimate LC3-II formation rates. As expected, suppressed increase in LC3-II by BafA1 treatment was shown in cells derived from Patient 1 when compared to the healthy controls (Figure 3b). Finally, to determine whether the autophagy pathway is disturbed upstream of WDR45, we examined levels of Beclin1, a protein that regulates the initial phase of the autophagosome formation. Fibroblasts from Patient 1 showed similar levels of Beclin1 to fibroblasts from healthy controls (Figure 3c) suggesting that the first part of autophagosome formation is unchanged and that disrupted autophagy occurs only in the later WDR45-mediated phases of the process.

### 2.4. Loss of WDR45 Is Linked to Disrupted Iron Recycling

To better understand how abnormalities in autophagy, lysosomal and mitochondrial dysfunction are linked to iron accumulation in WDR45 deficiency, we next investigated markers of iron homeostasis and recycling. To investigate whether the WDR45 missense mutation behaves similarly to a previously characterized WDR45-truncating mutation, we also included fibroblasts from another patient (Patient 2, L-8172, *WDR45*:c.519+1_3del) [9]. Abnormalities of autophagy, and lysosomal and mitochondrial dysfunction, reported here in Section 2.1, Section 2.2 and Section 2.3, have previously been shown for Patient 2 [9].

**Figure 4:**
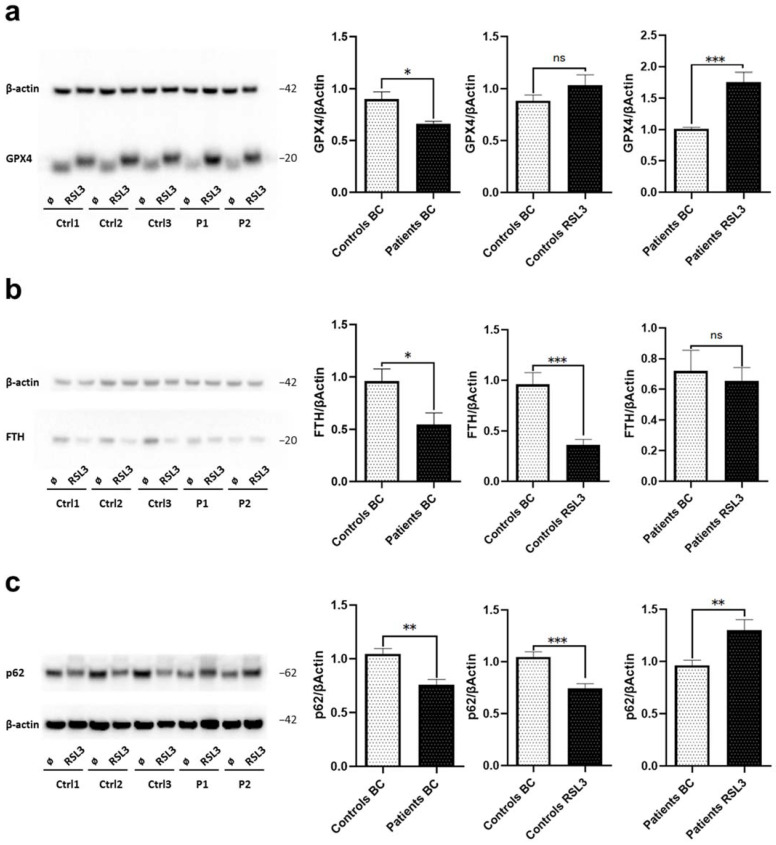
**Loss of WDR45 is linked to disrupted iron recycling via ferroptosis.** Western blot analysis of total protein extract from fibroblasts from: (**a**) Patients’ fibroblasts (P1, P2) and three healthy controls (Ctrl1, Ctrl2, Ctrl3) with antibodies against GPX4 and β-actin (loading control) under basal conditions and upon treatment with 1.5 µM of RSL3 for 6 h. (**b**) WDR45 mutation carriers (Patients, P1 and P2) and three healthy controls (Ctrl1, Ctrl2, Ctrl3) with antibody against FTH and β-actin (loading control) under basal conditions and upon treatment with 1.5 µM of RSL3 for 6 h. (**c**) Patients’ fibroblasts and three healthy controls with antibodies against p62 and β-actin (loading control) under basal conditions (BC) and upon treatment with 1.5 µM of RSL3 for 6 h. Data analysis was carried out for all Western blots with Ctrl1 in basal conditions set as 1 (first and second diagrams) or P1 in basal conditions (third diagram). The error bars indicate the standard error of the mean of n ≥ 3 independent experiments. Statistical significance was analyzed by Unpaired *t*-test (ns refers to *p* ≥ 0.05 (not significant); * *p* < 0.05; ** *p* < 0.01; *** *p* < 0.001).

Since increased cellular iron levels cause lipid peroxidation leading to oxidative stress and consequential iron-dependent cell death (ferroptosis), we first examined protein levels of markers of ferroptosis. We used the compound Ras-selective lethal small molecule 3 [(1S, 3R)-RSL3, RSL3], which is an inducer of ferroptosis through the inactivation of glutathione peroxidase 4 (GPX4), a regulator of ferroptosis [24,25,26,27]. Western blot analyses revealed that under basal conditions, protein levels of GPX4 in both WDR45-mutant fibroblast lines are decreased compared to the untreated healthy controls showcasing a possible abnormality in the antioxidant defense mechanism in Patients 1 and 2 (Figure 4a). Upon RSL3 treatment, we observed an exaggerated increase in GPX4 expression (with a slightly higher molecular weight possibly due to conformational changes [26]) in WDR45-mutant fibroblasts as opposed to the expected slightly increased expression in the controls (Figure 4a). These different response levels to RSL3 indicate differences in ferroptosis in mutant cells compared to healthy controls. Next, we examined in *WDR45*-mutant fibroblasts the level of ferritin heavy chain (FTH) that usually contributes to the safe sequestration of iron ions by immunoblotting. This analysis revealed decreased FTH protein levels in both patients compared to healthy controls under basal conditions (Figure 4b). Additionally, after RSL3 treatment, FTH protein levels decreased in healthy controls, as opposed to the unchanged levels in *WDR45*-mutant fibroblasts (Figure 4b). This indicates that induction of ferroptosis does not affect already altered FTH levels in *WDR45*-mutant fibroblasts.

Sequestosome1/p62 is a substrate for autophagic degradation; its gene inhibition significantly reduces cell viability and increases cellular lipid ROS levels [28,29], and it is also involved in the p62-Keap1-NRF2 antioxidant system participating in the RSL3 resistance mechanism [30]. As a marker of autophagic and ferroptotic clearance, we examined p62 protein levels by immunoblotting. Under basal conditions, we observed decreased levels of p62 in both *WDR45*-mutant fibroblast lines compared to healthy controls (Figure 4c). After RSL3 treatment, levels of p62 in *WDR45*-mutant fibroblasts from both patients increased in comparison to the same cells before treatment. In contrast, the levels of p62 decreased in all healthy controls after treatment, given the selective autophagy degradation triggered by p62’s RSL3 resistance mechanism [31] (Figure 4c). This further confirms the alterations in ferroptosis and autophagy in *WDR45*-mutant cells compared to healthy controls.

**Figure 5:**
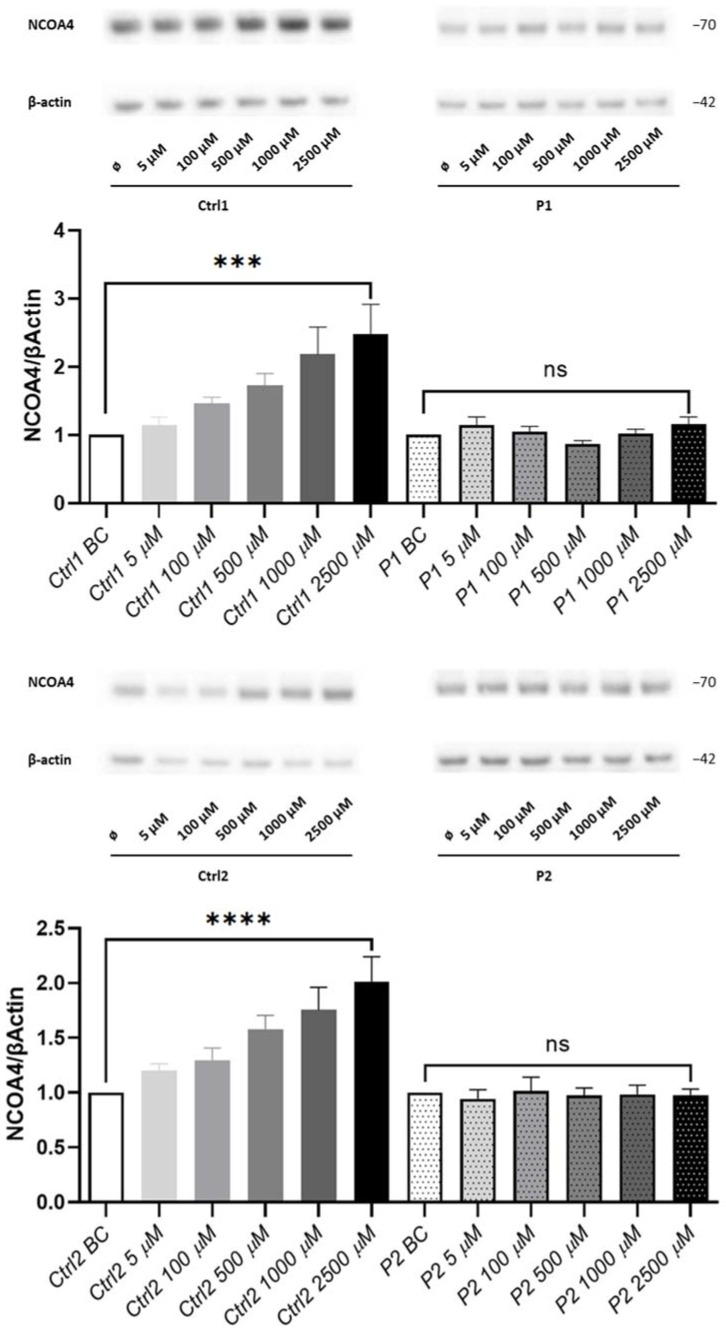
**Loss of WDR45 is linked to disrupted iron recycling via ferritinophagy.** Western blot analysis of total protein extract from fibroblasts from: WDR45 mutation carriers (P1 and P2) and two healthy controls (Ctrl1 and Ctrl2) with antibody against NCOA4 and β-actin (loading control) under basal conditions (BC) and upon treatment with 5, 100, 500, 1000 and 2500 µM of FAC for 24 h. Data analysis was carried out for all Western blots with Ctrl1 BC and P1 (for the upper panel) and Ctrl2 BC and P2 (for the lower panel) set as 1. The error bars indicate the standard error of the mean of n ≥ 3 independent experiments. One-way ANOVA analyzed statistical significance with post-hoc Turkey test (ns refers to *p* ≥ 0.05 (not significant); *** *p* < 0.001 and **** *p* < 0.0001 refers to significantly increased NCOA4 protein levels in fibroblasts treated with 2500 µM of FAC compared to the same but untreated cells (BC)).

Finally, we hypothesized that *WDR45* mutations might also cause deficits in ferritinophagy. Alterations in ferritinophagy contribute further to ferroptosis initiation through ferritin degradation, which triggers labile iron overload, lipid peroxidation, membrane impairment, and cell death [14,15,16]. Therefore, we examined the protein levels of the ferritinophagy marker NCOA4 [11] in fibroblasts from both patients and healthy controls treated with the iron donor ferric-ammonium citrate (FAC). We observed a gradient of increasing NCOA4 protein levels in healthy controls with increasing doses of FAC, as opposed to the unchanged NCOA4 protein levels in both *WDR45*-mutant cells (Figure 5). This result indicates increased levels of a cargo receptor for ferritin NCOA4 as a reaction to the abundance of iron ions in healthy controls, and alterations in this cellular response in patient’s cells.

## 3. Discussion

Corresponding to the broad spectrum of phenotypes seen in BPAN, the cellular consequences of mutant WDR45 are manifold, including autophagy defects, malfunctioning mitochondria, and endoplasmic reticulum stress, implying that WDR45 protein functions in diverse pathways directly or indirectly regulate these processes. However, observed phenomena like abnormalities in iron-containing organelles (lysosomes and mitochondria), autophagosome formation (which impacts ferritinophagy), and altered iron storage/capacity (which impacts ferroptosis) have not yet been arranged in a causal, functional, and temporal sequence. Thus, the exact molecular pathway of the disease remains elusive.

**Figure 6:**
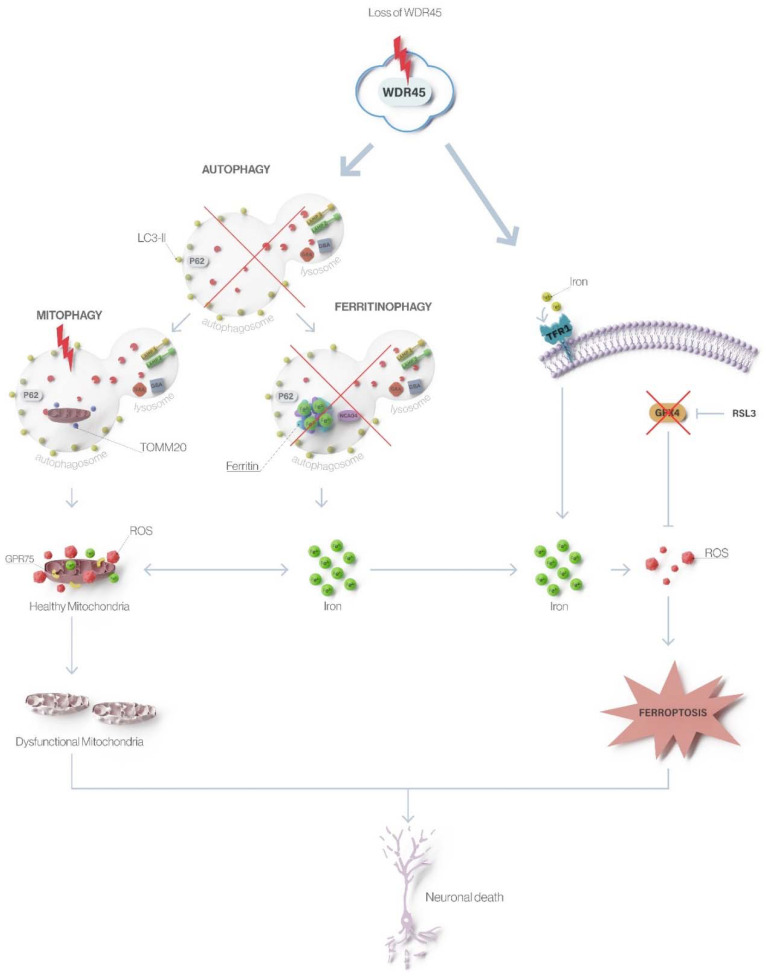
Loss of WDR45 leads to altered autophagy, ferritinophagy, and ferroptosis in β-propeller protein-associated neurodegeneration. Mutations in *WDR45* cause impaired autophagy as the primary defect that further leads to disturbed degradation of iron-rich ferritin (affecting ferritinophagy) and iron-containing organelles like mitochondria (affecting mitophagy). Lack of ferritinophagy leads to increased total cellular iron levels, which, through ROS formation and oxidative stress, triggers ferroptosis resulting in neuronal death.

We here extended previously reported molecular effects [8,9] on mitochondrial and lysosomal integrity and autophagy caused by low levels of functional WDR45 (Figure 6). Fibroblast cells from Patient 1 and Patient 2, carrying a missense and a truncating mutation, respectively, display many overlapping molecular phenotypes (this report and reference [9]). Thus, the missense mutation in Patient 1 likely also acts through a loss-of-function pathogenic mechanism. Specifically, decreased lysosomal markers LAMP1 and LAMP2 protein levels in *WDR45*-mutant fibroblasts suggested that elevated lysosomal iron may lead to loss of lysosomal integrity [32]. In addition, we observed significantly decreased protein levels of two crucial enzymes for lysosomal storage, GBA and GAA. The GBA depletion has been known to cause dysfunctional autophagy by inhibiting the lysosomal clearance of autophagic cargo [21]. Moreover, the depletion of GBA has also been known to trigger dysfunction of mitochondria (the main cellular storage organelles for iron besides lysosomes) by inhibiting mitochondrial priming, a critical step in the process of mitophagy [23]. We here showed an altered mitochondrial network and decreased TOMM20 protein levels in mutant fibroblasts indicating disrupted mitochondrial integrity that may be caused by an excess of redox-active iron and the resulting ROS generation [33] or by the inhibition of mitophagy [23]. These data additionally support the hypothesis that *WDR45* mutations lead to autophagic defects resulting in abnormalities in iron-containing organelles. In line with this, we confirmed altered lysosome-dependent autophagosome degradation by a comparable outcome of blocked autophagic flux in investigated cells as previously shown in other patients [2,9]. Accordingly, decreased LC3-II levels in *WDR45*-mutant patient’s fibroblasts in the presence and absence of BafA1 indicated impaired autophagosome maturation. To determine whether the autophagy pathway is disturbed before WDR45 is involved in the process, we analyzed the levels of Beclin1 protein since it regulates the initial phase of the autophagosome formation as well as the recruitment of subsequent factors needed for membrane elongation of the phagophore. As anticipated, Beclin1 protein levels did not differ between mutant and wild-type fibroblast lines. The data indicate that autophagy is blocked at an intermediate step during autophagosome formation, where WDR45 plays an important role.

Based on our observations, we hypothesized that dysfunctional WDR45 might cause deficits in autophagy-like ferritinophagy and, thereby, contributing to disrupted iron homeostasis and ferroptosis, resulting in neurodegeneration in BPAN [34]. We first focused on the reparative antioxidant GPX4 system, which pivotally controls ferroptosis and decreases the intracellular ROS level [25,27,35,36]. Enzymatic activity of GPX4 is vital to cells, given that the up-regulation of the enzyme can reduce phospholipid hydroperoxides, decrease oxidative stress and suppress ferroptosis [24,25,27,35,37,38,39]. Of note, mitochondria have been indicated to play a role in the deregulated lipid peroxidation caused by GPX4 deficiency [40]. Interestingly, as previously reported, *WDR45*-mutant fibroblasts from both patients showed decreased levels of GPX4 compared to healthy controls [41], suggesting an overall dysfunction in the GPX4 antioxidant protective system against oxidative stress caused by iron accumulation and signature of ferroptosis [42]. Additionally, we observed decreased FTH protein levels in the patients when compared to healthy controls, which is consistent with previous results [41], indicating disturbed iron storage in patient’s fibroblasts, leading to increased free iron accumulation. We next used RSL3, which leads to inactivation of GPX4 and subsequent elevation of lipid-ROS resulting in ferroptosis induction [26]. Upon binding to 14-3-3ε, GPX4 undergoes a conformational shift that makes it more prone to react with RSL3, which will then be able to inactivate the enzyme [26]. The treatment with this ferroptosis inductor significantly increased protein levels of GPX4 in both *WDR45*-mutant fibroblasts compared to the same cells under basal conditions, suggesting the susceptibility of WDR45 depleted cells. Accordingly, the RSL3 treatment caused decreased FTH protein levels in healthy control fibroblasts but not in *WDR45*-mutant fibroblasts, indicating that the induction of ferroptosis cannot ameliorate already altered FTH levels in WDR45 deficiency. This shortage of ferritin in *WDR45*-mutant cells may be one of the factors able to induce ROS and sensitize cells to ferroptosis.

Sequestosome1/p62 binds LC3, thus serving as a selective substrate for autophagic degradation [29]. Therefore, we examined protein levels of p62 as a marker of autophagic clearance and observed reduced expression in patients’ derived fibroblasts compared to controls. Since p62 is involved in the p62-Keap1-NRF2 antioxidant system participating in the RSL3 resistance mechanism, we next examined p62 protein levels upon RSL3 treatment also as a marker of ferroptotic clearance. After RSL3 treatment, levels of p62 in the *WDR45*-mutant fibroblasts increased compared to the untreated *WDR45*-mutant fibroblasts and, in contrast, decreased in all RSL3 treated healthy controls when compared to the not treated healthy fibroblasts. Altogether, our data suggest a significant impairment in these connected major stress response pathways (selective autophagy as clearance machinery and the GPX4 antioxidant system as a protective mechanism against iron-induced oxidative stress).

Lastly, we examined NCOA4, a marker of ferritinophagy in patient and control cells. Importantly, we report here for the first time unchanged levels of the ferritinophagy marker NCOA4 in FAC-treated *WDR45*-mutant fibroblasts as opposed to healthy controls showing increasing expression of NCOA4 directly proportional to the amount of FAC administered to the cells. These results indicate altered ferritinophagy and subsequent disruption in iron recycling as essential mechanisms in BPAN. Of note, NCOA4 has recently been unraveled as a critical player in an alternative autophagy-independent lysosomal transport pathway for ferritin [43], and NCOA4 itself seems to function as an iron-binding protein through residues 383–509 [13], confirming the importance of NCOA4 in iron homeostasis.

## 4. Materials and Methods

### 4.1. Subjects

We included fibroblast cultures from two female patients with BPAN (a new patient [Patient 1, L-11474] and a previously published patient [Patient 2, L-8172, *WDR45*:c.519+1_3delGTG; NM_007075.3] [9]) and three healthy, mutation-negative individuals (L-10180, L-3365, L-2153). The new patient underwent a detailed clinical examination, MRI, and Sanger sequencing of the entire *WDR45* coding region. The local ethics committee of the University of Lübeck approved the study, and all participants gave written informed consent for molecular analyses.

### 4.2. Cell Culture

Primary dermal skin fibroblasts were maintained in Dulbecco’s modified Eagle’s medium (DMEM, Thermo Scientific, Waltham, MA, USA) supplemented with 10% fetal bovine serum (Life technologies) and 1% penicillin/streptomycin (Life Technologies). Cells were cultivated at 37 °C and 5% CO_2_ in a humidified atmosphere. To assay cells upon autophagy inhibition, cells were treated with 10 nM of Bafilomycin A1 (Tocris Bioscience, Bristol, UK) for 6 h. To assay cells upon ferroptosis activation, cells were treated with 1.5 µM of RSL3 (Sigma-Aldrich, St. Louis, MO, USA) for 6 h. To evaluate iron clearance, cells were treated with 5, 100, 500, 1000, and 2500 µM of FAC (Sigma-Aldrich) for 24 h.

### 4.3. Sanger Sequencing and Quantitative PCR Analysis

Genomic DNA was isolated from fibroblast cultures using the DNeasy kit (Qiagen, Hilden, Germany). Sanger sequencing was used to screen for *WDR45* mutations. Total RNA from fibroblast cultures was prepared using the RNeasy kit (Qiagen) according to the manufacturer’s instructions. Oligo(dT) nucleotides from the Maxima First Strand cDNA Synthesis Kit (Thermo Scientific) served as primers for the synthesis of the complementary DNA (cDNA) by use of reverse transcriptase (RT). cDNA of *WDR45* was sequenced to investigate the expression of the mutated and wild-type alleles. In addition, we performed quantitative PCR analysis with SYBR Green (Roche Diagnostics, Basel, Switzerland) on a LightCycler480 (Roche Diagnostics) to examine the levels of *WDR45* cDNA in the patients compared to the controls. The housekeeping genes *ACTB* and *YWHAZ* were used as reference genes for normalizing the expression data.

### 4.4. Western Blot Analysis

Cell pellets were extracted using SDS extraction buffer (50 mM Tris–HCl pH 7.6, 150 mM NaCl, 1% DOC, 1% NP-40, and 0.1% SDS) or 1% Triton X-100 lysis buffer (containing 10% glycerol, 150 mM NaCl, 25 mM Hepes pH 7.4, 1 mM EDTA, 1.5 mM MgCl_2_, proteinase inhibitor cocktail) and gels were blotted onto nitrocellulose membranes. Antibodies used for immunoblotting were as follows: anti-LAMP (Lysosome-associated membrane glycoprotein)-1 (1:1000; Santa Cruz), anti-LAMP-2 (1:1000; Santa Cruz), anti-GBA (Lysosomal acid glucosylceramidase; 1:1000; Abcam), anti-GAA (Lysosomal alpha-glucosidase; Invitrogen; 1:1000), anti-TOMM20 (translocase of the outer mitochondrial membrane 20; 1:1000; Santa Cruz), anti-LC3A/B (microtubule-associated protein 1 light chain 3; 1:500; Cell Signaling), anti-Beclin 1 (1:1000, Cell Signaling), anti-Sequestosome1 (p62; 1:1000; Cell Signaling), anti-GPX4 (Glutathione peroxidase 4; 1:1300; Abcam), anti-ferritin heavy chain (FTH, 1:600; Cell Signaling), anti-NCOA4 (1:500; Bethyl Laboratories), and anti-β-actin (1:1,000,000; Sigma).

### 4.5. Immunofluorescence Staining and Image Analysis

For immunocytochemical analysis, cells on coverslips were fixed in 4% formaldehyde for 15 min, permeabilized, and blocked with 0.1% Triton X-100 in 4% normal goat serum in PBS for 1 h. Mitochondrial network interconnectivity was analyzed in cells stained for mitochondrial GRP75. Immunofluorescence staining was performed with primary antibody against GRP75 (1:1000; Abcam) and respective secondary fluorescence antibody (1:400; Life technologies). The images were taken as z-stacks by using a confocal microscope. The form factor was calculated using the formula [P_m_^2^]/[4πA_m_]), with P_m_ being the length of the mitochondrial outline and A_m_ being the area of the mitochondrion. At least ten cells from two coverslips per individual were analyzed, and a mean form factor was calculated as a measure for mitochondrial interconnectivity by using ImageJ (NIH software; Rasband, W.S., ImageJ, U. S. National Institute of Health, Bethesda, Maryland, USA, https://imagej.nih.gov/ij/, 1997–2018) as described previously [44].

### 4.6. Statistical Analysis

Differences were analyzed using unpaired *t*-tests or analysis of variance (ANOVA) with a Bonferroni–Dunn post-hoc test or a Turkey post-hoc test. The error bars indicate the standard error of the mean (SEM) of n ≥ 3 independent experiments.

## 5. Conclusions

Altogether our data suggest that dysfunctional WDR45 leads to autophagic defects resulting in disrupted iron recycling and storing, which is accompanied by alterations in iron-containing organelles (lysosomes and mitochondria) and mechanisms involved in iron recycling (ferritinophagy and ferroptosis) (Figure 6).

## Data Availability

The data presented in this study are available on request from the corresponding author.

## Supplementary Materials

The supporting information can be downloaded at: https://www.mdpi.com/article/10.3390/ijms23179524/s1.
